# MIND THE GAP: FINDINGS FROM THE AGE-FRIENDLY OXFORD COMMUNITY NEEDS ASSESSMENT

**DOI:** 10.1093/geroni/igz038.937

**Published:** 2019-11-08

**Authors:** Meghan A Young, Usha Dhakal, Valerie L Kessler, Suzanne R Kunkel

**Affiliations:** 1 Scripps Gerontology Center - Miami University, Oxford, Ohio, United States; 2 Miami University Scripps Gerontology Center, Oxford, Ohio, United States

## Abstract

In November 2017, Oxford, OH joined the AARP network of Age-Friendly Communities (AFCs). The first step in building an action plan through the AARP process is conducting a community needs assessment. Scripps Gerontology Center adapted the AARP Community Survey Questionnaire and mailed surveys to a random sample of 700 Oxford residents aged 50 years and older. The response rate was 46.8%. For seven of the eight domains of livability, individuals were asked how important is it to have particular services in the community (Likert scale) and whether the community provides the services (response options: yes, no, not sure). The responses to these questions were used to calculate a perceived gap score. The purpose of this project was to identify which domains had the largest perceived gaps, then further analyze individual item gaps. The three domains with the largest gaps were transportation (50.6%), housing (47.7%), and health (46.2%). Further analysis of the 60 individual domain items provided information about the type of gap. For example, 84% of respondents found the item “affordable public transportation” important. However, of those who said it is important, 73% perceived a gap in service provision, and 64% of the gap was due to not knowing if Oxford provides it. Communities may interpret a “not sure” gap as an opportunity to restructure how they promote services to older individuals. Implications of this research include proposing different ways of analyzing needs assessment data so AFCs can make efficient and effective changes for older adults to age in place.

